# Broadband circular polarizer for randomly polarized light in few-layer metasurface

**DOI:** 10.1038/s41598-019-38948-2

**Published:** 2019-02-22

**Authors:** Sang-Eun Mun, Jongwoo Hong, Jeong-Geun Yun, Byoungho Lee

**Affiliations:** Inter-University Semiconductor Research Center and School of Electrical and Computer Engineering, Seoul National University, Gwanak-Gu Gwanakro 1, Seoul, 08826 Korea

## Abstract

Controlling the polarization state of light has been a significant issue for various integrated optical devices such as optical imaging, sensors, and communications. Recent advances in metamaterials enable the optical elements for controlling light to be miniaturized and to have various multi-functions in subwavelength scale. However, a conventional approach of a circular polarizer has an inherent limitation to eliminate the unwanted circular polarization, which means that the efficiency varies significantly depending on the polarization state of incident light. Here, we propose a novel concept of a circular polarizer by combining two functions of transmission and conversion for orthogonal circular polarizations with a total thickness of 440 nm. The proposed three-layer metasurface composed of rotating silver nanorods transmits the left-handed circularly polarized (LCP) light with maintaining its own polarization state, whereas the right-handed circularly polarized (RCP) light is converted into LCP light. Regardless of the polarization state of incoming light, the polarization of light in the last medium is LCP state in the broadband operating wavelength range from 800 nm to 1100 nm. The converted RCP and the transmitted LCP have efficiencies of up to 48.5% and 42.3%, respectively. Thus the proposed metasurface serves as a stable circular polarizer for a randomly polarized light. In addition, high-efficiency asymmetric transmission of about 0.47 is achieved at the same time due to the conversion characteristic of RCP component. The proposed metasurface has the significance as an ultra-thin optical element applicable to optical switching, sensors, and communications in unidirectional channel as well as a broadband circular polarizer for randomly polarized light.

## Introduction

Polarization is a unique nature of light that cannot be distinguished by human eyes. Controlling the polarization of light has been applied to various optical systems such as optical sensing, imaging, and communication^1,2^. Especially, the circularly polarized lights, which are distinguished by left-handed circularly polarized (LCP) and right-handed circularly polarized (RCP) lights, interact differently with chiral media depending on their handedness. Then, it is accompanied by unique characteristics such as bianisotropy, magneto-electric coupling, and circular dichroism^3–5^, which provide importance for applications to biological molecules and future display systems. Conventional approaches to manipulate the circular polarization state of light are to utilize the combination of linear polarizers and waveplates or Bragg reflection of a cholesteric liquid crystal^6–9^. However, optical devices based on these conventional methods have disadvantages of a bulky size, a narrow operating frequency range, and an inherent absorption, which limit integration into very small form factors of today’s optical systems.

**Figure 1:**
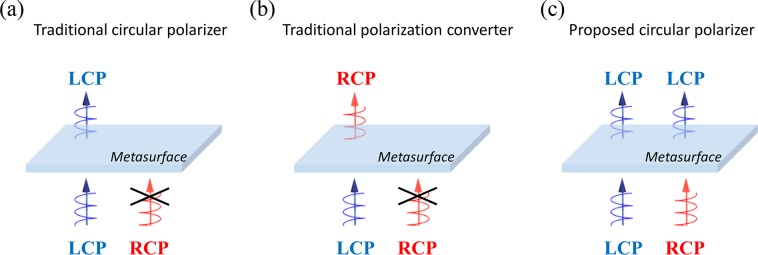
Schematic illustrations of a conventional (**a**) circular polarizer and (**b**) polarization converter. (**c**) A schematic diagram of the proposed circular polarizer combining transmission and conversion functions for each circular polarization.

Recently, plasmonic metamaterials or metasurfaces, composed of periodic artificial nanostructures with subwavelength unit cells, have attracted much attention since they provide a new way to design integrated optical devices for polarization control with subwavelength thickness, broad operating bandwidth, and simple fabrication process^3–5,10–20^. Many types of circular polarizers have been realized using three-dimensional (3D) chiral metamaterials^12–15^, two-dimensional (2D) chiral metasurfaces^16–19^, and single layer metasurfaces^20,21^ in wide frequency regime including GHz, THz, and optical range. Since planar structure has a weak circular dichroism along the propagation direction, single layer metasurfaces have implemented circular polarizers at single frequency almost in GHz range. Although studies for polarization control based on metamaterials or metasurfaces have made significant advances in conventional optical devices, circular polarizers based on the consistent handedness of chirality still have inherent limitations that reflect the undesired circular polarization component as shown in Fig. 1(a). Thus, the maximum efficiency of the circular polarizers cannot exceed half for un-polarized incident light and they cannot maintain consistent efficiency depending on the polarization state of incident light.

Also, polarization control techniques such as polarization converters (linear to linear^22–27^, linear to circular^28,29^, circular to circular^30–32^) have been studied as an essential optical device for manipulating the characteristics of light. Various types of polarization converters using linear or circular polarizations have been implemented in reflection^22,23^ and transmission modes^24–31^. Recently, it has been demonstrated that anisotropy of few-layer metasurfaces enables high-performance polarization conversion accompanied by asymmetric transmission of light^24,31–33^. However, similar to the case of the conventional circular polarizer, polarization converters only increase the ratio of the converted component while little consideration is given to the unwanted orthogonal polarization component as shown in Fig. 1(b). In particular, since the transmission characteristics of the circularly polarized light depends on the consistent handedness of the 3D or 2D chiral structures, the conventional converter scheme has limitations in implementing different functions for orthogonal circular polarization states.

In this paper, we propose a novel concept of broadband circular polarizer based on few-layer anisotropic metasurface by combing these two concepts of a transmissive polarizer and converter. The proposed metasurface is composed of three-layer rotating silver nanorods with inconsistent 3D chirality that provides distinct functions for two orthogonal circular polarizations. It operates as a transmissive polarizer for certain circular polarization of incident light with the inherent polarization state maintained, while it acts like a polarization converter for orthogonal circular polarization. We verified the functionality of the few-layer metasurface via numerical simulation as well as theoretical calculation. Therefore, the proposed device becomes a novel circular polarizer that maintains a stable function regardless of the polarization state of incident light as shown in Fig. 1(c). In addition, high-efficiency asymmetric transmission of circular polarized light is accompanied due to the anisotropic property of the proposed metasurface.

### Metasurface design

**Figure 2:**
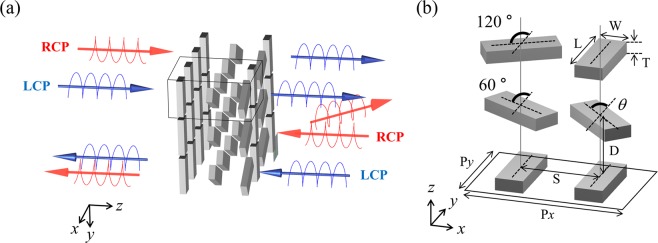
(**a**) Illustration of the designed metasurface composed of rotating silver nanorods. The LCP component is transmitted with maintaining its own polarization and RCP is converted into orthogonal polarization in forward propagation, which can serve as a high efficiency circular polarizer regardless of the incident polarization. The asymmetric transmission can be obtained in the other direction. (**b**) Unit cell structure of three-layer metasurface with geometric details including rotation angles of each nanorod. The length (L), width (W), and thickness (T) are set to 235 nm, 90 nm, and 40 nm, respectively. The separation distance (D) between each layer is 200 nm and the distance (S) between two sub-unit cells is 255 nm. The periods of unit cell are P*x* = 510 nm and P*y* = 255 nm in *x* and *y* directions.

The proposed concept for circular polarizer is presented in Fig. 2(a). For a forward propagation, an incident LCP light is transmitted with maintaining its polarization state and an incident RCP light is transformed into LCP light, while a backward incident light shows different optical responses due to the anisotropic feature of the proposed metasurface. A unit cell of three-layer metasurface is presented in Fig. 2(b) and it consists of two different sub-unit cells. The left one is composed of three silver nanorods having rotation angles of 0, 60, and 120 degrees with respect to *y*-axis, and the other on the right is composed of two parallel nanorods in the first and third layers and one nanorod with variable rotation angle *θ* in the second layer. The varying rotation angle can induce coupling by adjusting the gap distance between two sub-unit cells, which will be discussed further. The nanorods have dimensions of length L = 235 nm, width W = 90 nm, and thickness T = 40 nm. The separation distance between each layer is D = 200 nm, and periods of each unit cell in *x* and *y* directions are P*x* = 510 nm and P*y* = 255 nm, respectively, with the distance between two sub-unit cells S = 255 nm. The total thickness of the proposed metasurface is 440 nm, which is extremely thin. The numerical calculation for analyzing the proposed metasurface is carried out using the commercial tool (CST Microwave Studio). The material of nanorods is chosen as silver due to low intrinsic loss of which dispersion function is taken from Johnson and Christy^34^. For theoretical calculation, we set the background medium to air without a substrate. It is necessary to make the background medium homogeneous by depositing about 200 nm of the same material as the substrate to cover the top layer for high efficiency application and inhibition of oxidization. Periodic boundary conditions are set along *x* and *y* directions, and an open boundary is applied in *z* direction.

The designed metasurface is optimized through the following matrix analysis. The Jones matrix is used to determine the transmission characteristics for a forward incident light^35^. The incident and transmitted electric fields are related by complex Jones matrix *T* ^*f*^ expressed as

1 $$[\begin{array}{c}{T}_{LCP}\\ {T}_{RCP}\end{array}]=[\begin{array}{cc}{T}_{LL}^{f} & {T}_{LR}^{f}\\ {T}_{RL}^{f} & {T}_{RR}^{f}\end{array}][\begin{array}{c}{I}_{LCP}\\ {I}_{RCP}\end{array}]={T}^{f}[\begin{array}{c}{I}_{LCP}\\ {I}_{RCP}\end{array}]$$

**Figure 3:**
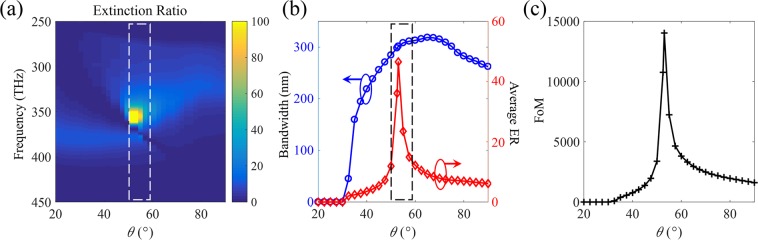
(**a**) The ER according to the varying rotation angle, *θ*, of one nanorod in the broadband frequency range. The calculated results of (**b**) operation bandwidth, average ER, and (**c**) FoM for the variable rotation angle. The highest value of FoM is achieved inside the region of black dashed line.

Thus, the LCP component of total transmitted light is written by

2 $$\begin{array}{c}{t}_{LCP}=|{T}_{LL}^{f}{|}^{2}{I}_{LCP}+|{T}_{LR}^{f}{|}^{2}{I}_{RCP},\\ {t}_{RCP}=|{T}_{RL}^{f}{|}^{2}{I}_{LCP}+|{T}_{RR}^{f}{|}^{2}{I}_{RCP},\end{array}$$

where superscript *f* means forward propagation. *I*_*LCP*_ and *I*_*RCP*_ represent the intensities of the incident LCP and RCP components, respectively, assuming that those are the same for numerical calculation. The proposed novel concept is to design a high efficiency circular polarizer more precisely using all four elements in Jones matrix, unlike conventional circular polarizers which ignore the off-diagonal elements (${T}_{RL}^{f}\approx {T}_{LR}^{f}\approx {\rm{0}}$). Therefore, the extinction ratio (ER) is defined as the ratio of LCP to RCP in total transmitted light, $ER=(|{T}_{LL}^{f}{|}^{2}+|{T}_{LR}^{f}{|}^{2})/(|{T}_{RL}^{f}{|}^{2}+|{T}_{RR}^{f}{|}^{2})$, including all four components in transmission matrix. To achieve high performance as a circular polarizer, the proposed structure should have a high value of the ER. Figure 3(a) shows the calculated ER according to the change of rotation angle *θ*. As the rotation angle *θ* increases, the ER tends to increase with the increasing bandwidth up to about 60 degrees, which is denoted with white dashed lines. Not only the ER but also operation bandwidth is an important factor of circular polarizers. We can calculate the operation bandwidth as the wavelength range where the ER is larger than 2. Therefore, the overall figure of merit (FoM) is defined by the multiplication of the averaged ER and the operation bandwidth. The average ER is the average value for the wavelength in the operating bandwidth. Figure 3(b) shows the operation bandwidth and the average ER according to the corresponding variable angle *θ* in order to optimize the proposed structure with high FoM. The highest value of FoM is achieved at the rotation angle of 53 degrees as shown in Fig. 3(c). The proposed metasurface is designed based on this argument, and the analysis of characteristics and principles will be followed in the next section.

## Results and Discussion

**Figure 4:**
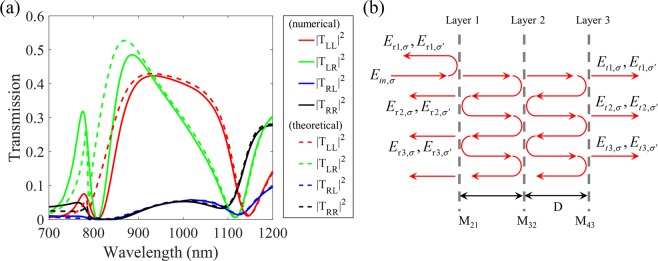
(**a**) Transmission spectra of the four elements in the transmission matrix for LCP and RCP incident lights. The solid and dashed lines indicate the numerical and theoretical results, respectively. (**b**) Scheme of the proposed three-layer metasurface for representing the multiple reflection and transmission for analysis using a wave transfer matrix method.

The transmission spectra for LCP and RCP incidences are shown in Fig. 4(a) with the comparison of numerical simulation and theoretical calculation at the condition of the highest FoM where the rotation angle *θ* is 53 degrees. The solid lines are numerical results and the dashed lines are theoretical results based on a wave transfer matrix method which will be discussed below. As expected from the results in Fig. 2, we found that the ${T}_{LL}^{f}$ and ${T}_{LR}^{f}$ components exhibit a similar high transmission over broad bandwidth from 800 nm to 1100 nm. The other coefficients (${T}_{RL}^{f}$ and ${T}_{RR}^{f}$), which are the unwanted RCP components in the transmitted end, show very low transmittance. This result means that the transmitted light is always LCP state regardless of the polarization state of the incoming circularly polarized light. Also, the maximum efficiencies for LCP and RCP incident lights are 42.3% and 48.5% at the almost same wavelength, respectively. The important point is not only to achieve a high FoM value, but also to show similar transmission and conversion efficiencies for both polarizations (*i.e*.${T}_{LL}^{f}\approx {T}_{LR}^{f}$). Here, we assume randomly polarized light as the light with a random distribution of the intensities of LCP and RCP, thereby excluding the interference effect on the random phase, since the phasor of random light is uniformly distributed between 0 and 2π and its average value is zero^36^. For randomly polarized light, the intensities of LCP and RCP are statistically 0.5 (*I*_*LCP*_ = *I*_*RCP*_ = 0.5), respectively. Even in the case of randomly polarized light in which the intensity of each circularly polarized light has a random distribution of values between 0 and 1, almost constant efficiency of LCP light is transmitted under this condition, ${T}_{LL}^{f}\approx {T}_{LR}^{f}$. That is, the proposed metasurface maintains stable function as a circular polarizer irrespective of the polarization state of incident light even though it is a randomly polarized light mixed with two circular polarization states.

Since we can regard the proposed structure as a Fabry-Pérot like metamaterial, a wave transfer matrix method is used for the theoretical calculation of multiple reflection and transmission in the few-layer metasurface as presented in Fig. 4(b) for the scheme. We obtain the transmission and reflection matrix elements for every layer from the simulation and calculate the scattering parameters for 4 by 4 matrix formulation denoted by *M*_*ba*_ matrices^22,32^. The propagation through a homogeneous medium (*b*) between each layer is described by the matrix *P*_*b*_. For simple analysis, we set the background medium to air. Then, we can determine the total *M* matrix for the proposed structure through each matrix multiplication in order, which is written as:

3 $$M={M}_{43}{P}_{3}{M}_{32}{P}_{2}{M}_{21}.$$

Finally, we retrieve the transmission coefficients from the overall *M* matrix considering that there is no backpropagation component after passing through layer 3 (see Supplementary information). The theoretically calculated result is shown in Fig. 4(a) with dashed lines, which is in good agreement with the numerically simulated result. Small discrepancies appear in the short wavelength region since the thickness of the nanorods is completely ignored in the transfer matrix method. However, these tiny errors are not significant and they are almost identical at wavelengths above 900 nm. Therefore, we can assert that the interpretation using the applied transfer matrix method is valid for the analysis of the proposed few-layer metasurface.

**Figure 5:**
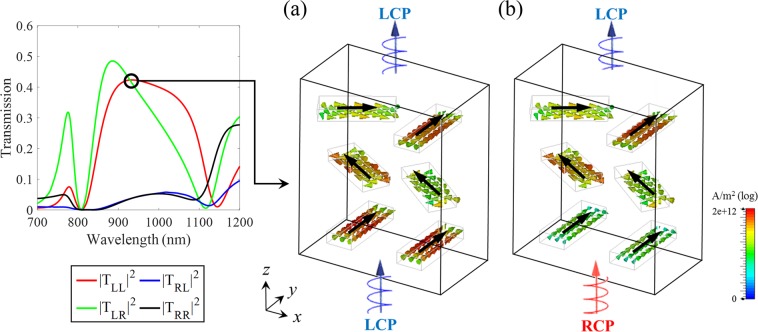
The induced current densities for (**a**) LCP and (**b**) RCP lights at the wavelength of 930 nm where the transmission is same for both incidences. By the coupling between two sub-unit cells, the current distributions induced by LCP and RCP lights are almost similar in the operating region. Consequently, the LCP state is transmitted for both incidences.

To further analyze the results, the induced current densities for LCP and RCP incidences are shown in Fig. 5 at the wavelength of 930 nm in the operating area. The LCP light passes the proposed metasurface almost transparently since LCP light has completely different handedness of rotating nanorods in two sub-unit cells as shown in Fig. 5(a). Therefore, the transmission spectrum ($|{T}_{LL}^{f}{|}^{2}$) is broader and smoother for LCP incident light in the operating region. On the other hand, RCP light tries to produce a resonance while rotating along forward *z* direction consistent with the handedness of one sub-unit cell on the left. However, this resonance condition is prohibited since the current distributions of the nanorods in the second and third layers are induced along almost parallel directions by strong coupling as shown in Fig. 5(b). Therefore, the polarization state of RCP is converted into the orthogonal handedness (i.e. LCP state) across the second-third layers. That is why the field distribution is almost similar whether the incident light is RCP or LCP state. This result implies that a novel concept is completely differentiated from the previous studies that proposed a circular polarizer which totally reflects unwanted polarization component.

**Figure 6:**
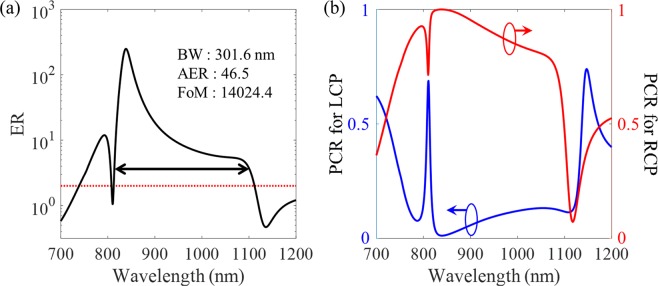
Numerically calculated results for the spectrum of (**a**) ER and (**b**) PCR for LCP and RCP, respectively. The broad bandwidth over 300 nm, the average ER of 46.5, and the FoM more than 14000 are achieved, simultaneously. The ratio values close to 0 and 1 are obtained for PCR for LCP and RCP lights, respectively, which means that RCP light is almost converted into orthogonal polarization and LCP light is transmitted through the proposed structure without any conversion.

To figure out the properties of the proposed structure as a circular polarizer combining the functions of transmission and conversion, we calculate the ER and the polarization conversion ratio (PCR) as shown in Fig. 6. The operation bandwidth where the ER is above the value of two as indicated with red dashed line is achieved over 300 nm and the FoM reaches about 14000 in the wavelength range from 800 nm to 1100 nm. In addition, the polarization conversion properties can be intuitively characterized by the indicator of PCR which is defined as:

4 $$PC{R}_{\sigma }=|{T}_{\sigma ^{\prime} \sigma }{|}^{2}/(|{T}_{\sigma ^{\prime} \sigma }{|}^{2}+|{T}_{\sigma \sigma }{|}^{2})$$

where *σ* and *σ´* mean the polarization states of the incident and transmitted lights, respectively. The condition for perfect polarization conversion is PCR = 1. The value of PCR for RCP light is close to 1 which means that RCP light has been almost converted to LCP light, while PCR for LCP light represents a value close to 0 which means that LCP light has transmitted through the proposed structure without any conversion, as shown in Fig. 6(b). These results through the indicators of ER and PCR verify the analysis of the transmission spectra for both of LCP and RCP incidences discussed above, and support that the proposed structure is a high performance circular polarizer in terms of the transmitted light although the efficiency is not quite high.

We have discussed the proposed structure as a broadband circular polarizer for randomly polarization light so far. Due to the structural anisotropy of the proposed metasurface, a unique characteristic of optical responses can be found in the backward propagating light. The Jones matrix for backward propagation along −*z* direction can be written as:

5 $$[\begin{array}{c}{T}_{LCP}\\ {T}_{RCP}\end{array}]=[\begin{array}{cc}{T}_{LL}^{b} & {T}_{LR}^{b}\\ {T}_{RL}^{b} & {T}_{RR}^{b}\end{array}][\begin{array}{c}{I}_{LCP}\\ {I}_{RCP}\end{array}]={T}^{b}[\begin{array}{c}{I}_{LCP}\\ {I}_{RCP}\end{array}].$$

Comparing Eq. (5) with Eq. (1) for forward propagation along +*z* direction, the elements of Jones matrix have the following relations by the reciprocity theorem since the proposed structure does not contain magneto-optic coupling effect.

6 $${({T}^{f})}^{T}={T}^{b},$$

7 $${T}_{LR}^{f}={T}_{RL}^{b},{T}_{LL}^{f}={T}_{LL}^{b}.$$

**Figure 7:**
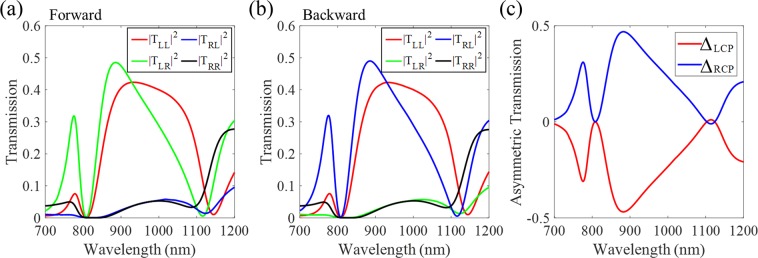
Numerical results of transmission spectra for (**a**) forward and (**b**) backward propagating lights, respectively. The cross-polarization components exhibit different optical responses depending on the direction of incident light. (**c**) The asymmetric transmission for LCP and RCP lights calculated from the numerical results in parts (**a**,**b**).

The numerical results of the transmission spectra for forward and backward propagation lights verify this relationship as shown in Fig. 7(a,b). Then, we calculate the asymmetric transmission which is defined as the difference between the transmissions of forward and backward propagating waves. Using above relation, the asymmetric transmission can be simply written as:

8 $${{\rm{\Delta }}}_{LCP}=({|{T}_{LL}^{f}|}^{2}+{|{T}_{RL}^{f}|}^{2})-({|{T}_{LL}^{b}|}^{2}+{|{T}_{RL}^{b}|}^{2})={|{T}_{RL}^{f}|}^{2}-{|{T}_{LR}^{f}|}^{2}=-\,{{\rm{\Delta }}}_{RCP}$$

The asymmetric transmission of LCP and RCP is presented in Fig. 7(c) and the high performance reaches the value of 0.47 at the wavelength of 883 nm. The highest asymmetric transmission is generated by the conversion property (cross-polarization) of the proposed structure, even if the transmission coefficients of co-polarization are large. We confirm that the results are not highly sensitive to changes in geometric parameters, alignment mismatches between layers, and incidence angle (see Supplementary information). The novelty of this results is explained by the fact that high asymmetric transmission can be achieved even in a planar metasurface where the handedness of chirality is not consistent (i.e. the rotation handedness changes along the propagation direction).

## Conclusion

In conclusion, we have proposed a novel circular polarizer with a few-layer metasurface composed of rotating silver nanorods in two sub-unit cells by combing the transmissive and convertive optical responses for circularly polarized light in the optical spectral range while having a thickness of 440 nm. We numerically demonstrate the simultaneous high transmission for LCP incidence and high conversion for RCP incidence with nearly similar efficiencies over the operation bandwidth from 800 nm to 1100 nm, which results in improving the efficiency of LCP light at the transmission end irrespective of the polarization state of the incident light. The performance of the proposed structure is verified by using the Jones matrix basis and the transfer matrix method, and we can confirm the validity of the interpretation by matching the theoretically calculated results. In addition, the anisotropy of the structure leads to not only polarization conversion but also asymmetric transmission characteristic with high efficiency at 883 nm. This result has great significance as a stable circular polarizer that operates independently for the polarization state of incident light. The proposed approach based on multi-functionality is expected to extend to the visible region and presents a new manner to overcome the efficiency limit of commercialized circular polarizers. Furthermore, it provides a new path of optical devices based on the metasurface for polarization manipulation with anisotropic property, which will be applied to practical applications including integrated photonic devices, optical sensors, and optical communications.

## Supplementary information

Broadband circular polarizer for randomly polarized light in few-layer metasurface_Supplementary information
