# Supplementary material for: Broadband circular polarizer for randomly polarized light in few-layer metasurface
Source: Sci Rep. 2019 Feb 22;9:2543. doi: 10.1038/s41598-019-38948-2 (PMC6384911; doi:10.1038/s41598-019-38948-2)
Supplement: Supplementary file 1 — Broadband circular polarizer for randomly polarized light in few-layer metasurface_Supplementary information [file 41598_2019_38948_MOESM1_ESM.doc]

**Supplementary Information**

Broadband circular polarizer for randomly polarized light in few-layer metasurface

Sang-Eun Mun, Jongwoo Hong, Jeong-Geun Yun, and Byoungho Lee^*^

Inter-University Semiconductor Research Center and School of Electrical and Computer Engineering, Seoul National University, Gwanak-Gu Gwanakro 1, Seoul 08826, Korea

*Corresponding author’s E-mail: byoungho@snu.ac.kr

**Part 1. Details of theoretical calculation using transfer matrix method**

Since the proposed structure has a sub-wavelength scale and the thickness of each layer is much smaller than the interlayer distance, theoretical approximation based on a transfer matrix method is well maintained. The proposed three-layer metasurface can be simplified as shown in Fig. S1. We can consider the multiple transmission and reflection components into one forward and one backward propagating electric fields in each medium^1,2^.

$\left(\begin{matrix} E_{b\sigma}^{f} \\ E_{b\sigma'}^{f} \\ E_{b\sigma}^{b} \\ E_{b\sigma'}^{b} \end{matrix}\right)=M_{ba}\left(\begin{matrix} E_{a\sigma}^{f} \\ E_{a\sigma'}^{f} \\ E_{a\sigma}^{b} \\ E_{a\sigma'}^{b} \end{matrix}\right),$ (S1)

$M_{ba}=\left(\begin{matrix} 1 & 0 & -r_{b\sigma,b\sigma} & -r_{b\sigma,b\sigma'} \\ 0 & 1 & -r_{b\sigma',b\sigma} & -r_{b\sigma',b\sigma'} \\ 0 & 0 & t_{a\sigma,b\sigma} & t_{a\sigma,b\sigma'} \\ 0 & 0 & t_{a\sigma',b\sigma} & t_{a\sigma',b\sigma'} \end{matrix}\right)^{-1}\left(\begin{matrix} t_{b\sigma,a\sigma} & t_{b\sigma,a\sigma'} & 0 & 0 \\ t_{b\sigma',a\sigma} & t_{b\sigma',a\sigma'} & 0 & 0 \\ -r_{a\sigma,a\sigma} & -r_{a\sigma,a\sigma'} & 1 & 0 \\ -r_{a\sigma',a\sigma} & -r_{a\sigma',a\sigma'} & 0 & 1 \end{matrix}\right),$ (S2)

where subscripts *a* and *b* represent the media before and after each layer, and subscripts *σ* and *σ´* denote the polarization states of light orthogonal to each other. Superscripts *f* and *b* mean forward and backward propagation, respectively. The propagation through a homogeneous medium with the separation distance D is described by the matrix *P_b_*, $P_{b}=\text{diag}(\text{exp}(ik_{0}n\text{D}),\text{exp}(ik_{0}n\text{D}),\text{exp}(-ik_{0}n\text{D}),\text{exp}(-ik_{0}n\text{D}))$, where *k*_0_ is the wave number in free-space and *n* is the refractive index in medium *b*. The overall transfer matrix through the whole layer is written as $M=M_{43}P_{3}M_{32}P_{2}M_{21}$. We can divide the *M* matrix into four 2 by 2 matrices as follows, *M_A_* = *M* (1:1:2, 1:1:2), *M_B_* = *M* (1:1:2, 3:1:4), *M_C_* = *M* (3:1:4, 1:1:2), and *M_D_* = *M* (3:1:4, 3:1:4). In the proposed concept, there are no back-propagating fields in the medium 4, $E_{4\sigma}^{b}=E_{4\sigma'}^{b}=0$. Then, the transmission and reflection fields for both orthogonal polarizations are determined as follows:

$\begin{matrix} \left(\begin{matrix} E_{4\sigma}^{f} \\ E_{4\sigma'}^{f} \end{matrix}\right)=M_{A}-M_{B}M_{D}^{-1}M_{C}\left(\begin{matrix} E_{1\sigma}^{f} \\ E_{1\sigma'}^{f} \end{matrix}\right) , \text{(transmission)} \\ \left(\begin{matrix} E_{1\sigma}^{b} \\ E_{1\sigma'}^{b} \end{matrix}\right)=-M_{D}^{-1}M_{C}\left(\begin{matrix} E_{1\sigma}^{f} \\ E_{1\sigma'}^{f} \end{matrix}\right) , \text{(reflection)} \end{matrix}$ (S3)

This approach can be extended to more layers and is useful in a few-layer metasurface analysis.

**
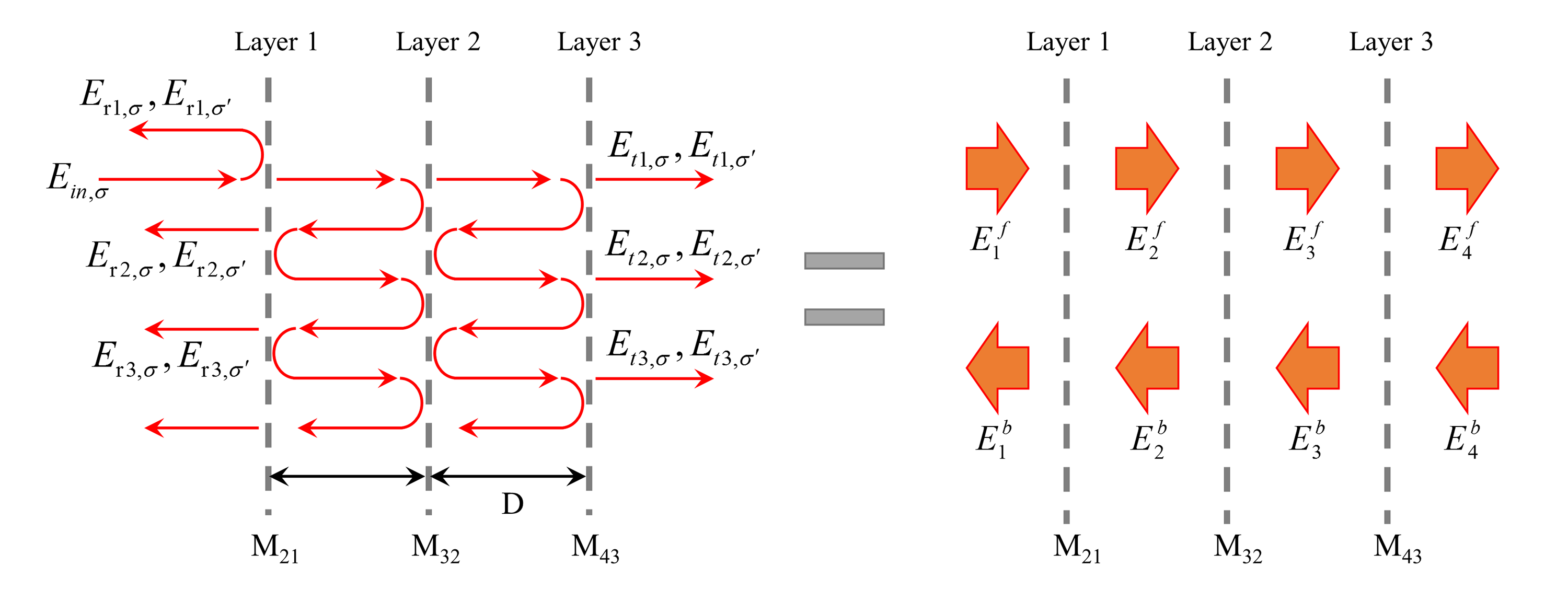
**

**Figure S1.** Scheme of the proposed three-layer metasurface for representing the multiple reflection and transmission and an equivalence scheme that simplifies it in each layer.

**Part 2. Influence of misalignment of each layer**

The proposed few-layer metasurface has the advantage of being robust to the misalignment of each layer. As the proposed concept is not based on the resonance of individual silver nanorods, the spatial shift in both *x* and *y* directions is not a sensitive factor to transmission and conversion if only the rod arrangement in each layer is well maintained. We verify this by shifting each of second and third layer and both layers compared to the case of ideal alignment as shown in Fig. S2.

**
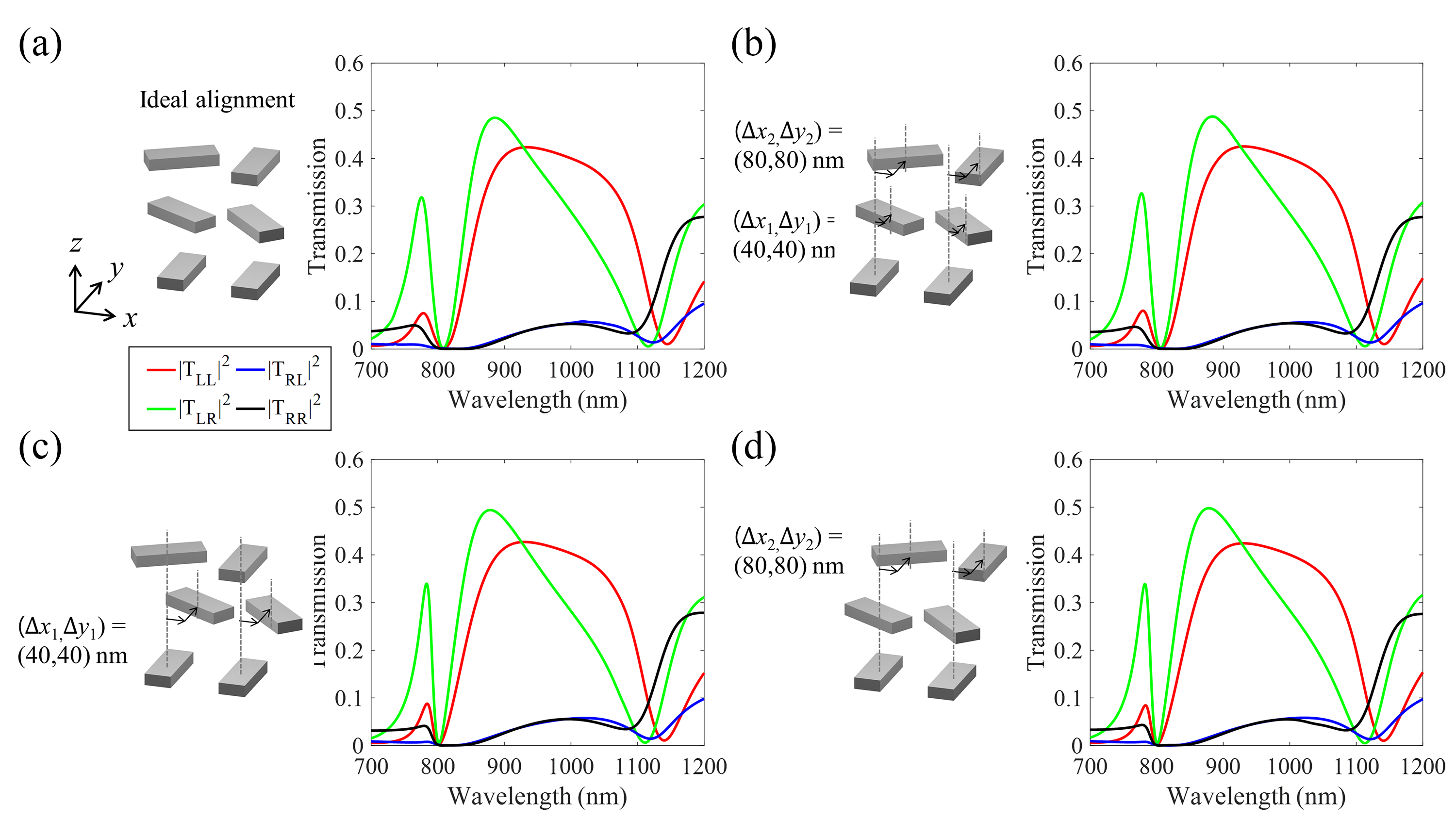
**

**Figure S2.** Transmission spectra corresponding to the case of (a) ideal alignment, (b) misalignment of the second and third layers, (c) misalignment of the second layer, and (d) misalignment of the third layer. The legends of all spectra are same as in (a). The $(\Delta x_{1},\Delta y_{1})$ and $(\Delta x_{2},\Delta y_{2})$ indicate shift variances of the second and third layers along *x* and *y* directions, respectively.

**Part 3. Influence of geometric parameters on the transmittance**

The proposed metasurface is composed of silver nanorods in two sub-unit cells. In the main text, we analyze the effect of rotation angle of nanorod to enhance the coupling between two sub-unit cells. The induced current distribution by the coupling is affected by the change in the geometric paramters of nanorods. As shown in Fig. S3, the transmission spectra according to the variation of geometric parameters of nanorod and the distance betweeen each layer are calculated through numerical simultions to optimize the proposed metasurface. As the geometric parameters change, we can see that $|T_{LL}|^{2}$ and $|T_{LR}|^{2}$ components are slightly affected. We choose optimal values of the length, the width, and the layer distance to be 235 nm, 90 nm, and 200 nm to achieve both high transmission and conversion for LCP and RCP lights.


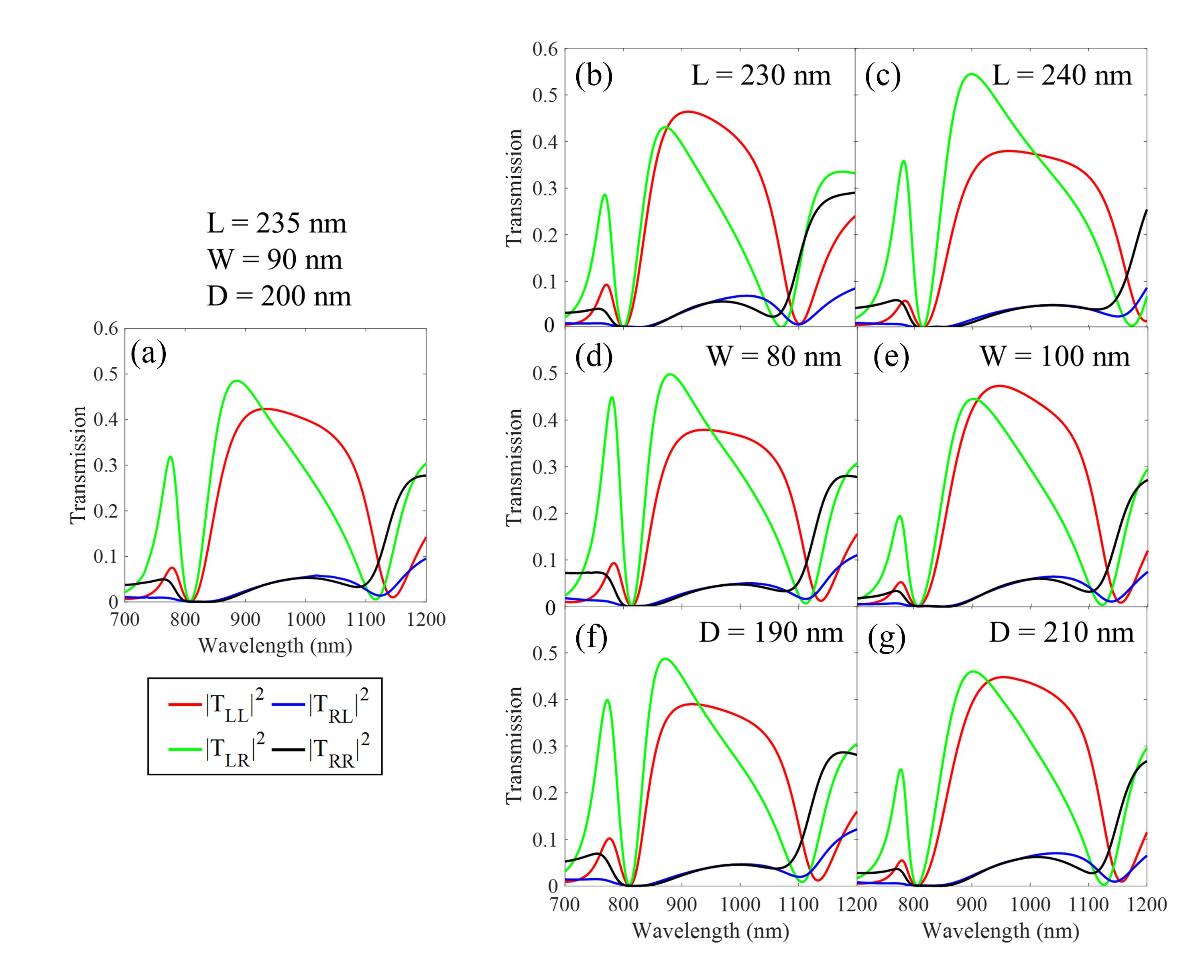


**Figure S3.** Transmission spectra for the variation of geometric parameters. (a) Transmission spectra for the designed metasurface with L = 235 nm, W = 90 nm, and D = 200 nm, which is analyzed in the main text. Transmission spectra according to the length (L) of (b) 230 nm and (c) 240 nm, the width (W) of (d) 80 nm and (e) 100 nm, and the layer distance (D) of (f) 190 nm and (g) 210 nm. The legends of all spectra are same as in (a).

**Part 4. Influence of oblique incidence on the transmittance**

Dealing with the problems of alignment, geometric parameter, and angular sensitivities is important for practical application. Here, we address the angular sensitivity issue of the proposed metasurface for oblique incidence. Due to the subwavelength thickness of each layer, oblique incidence does not make a large amount of additional phase accumulation. However, as the incident angle increases, it is inevitably accompanied by a reduction of transmission efficiency due to the change of inter-layer interaction. As shown in Fig. S4, the proposed metasurface exhibits a relatively high tolerance with respect to the incidence angle within about 20 degrees.


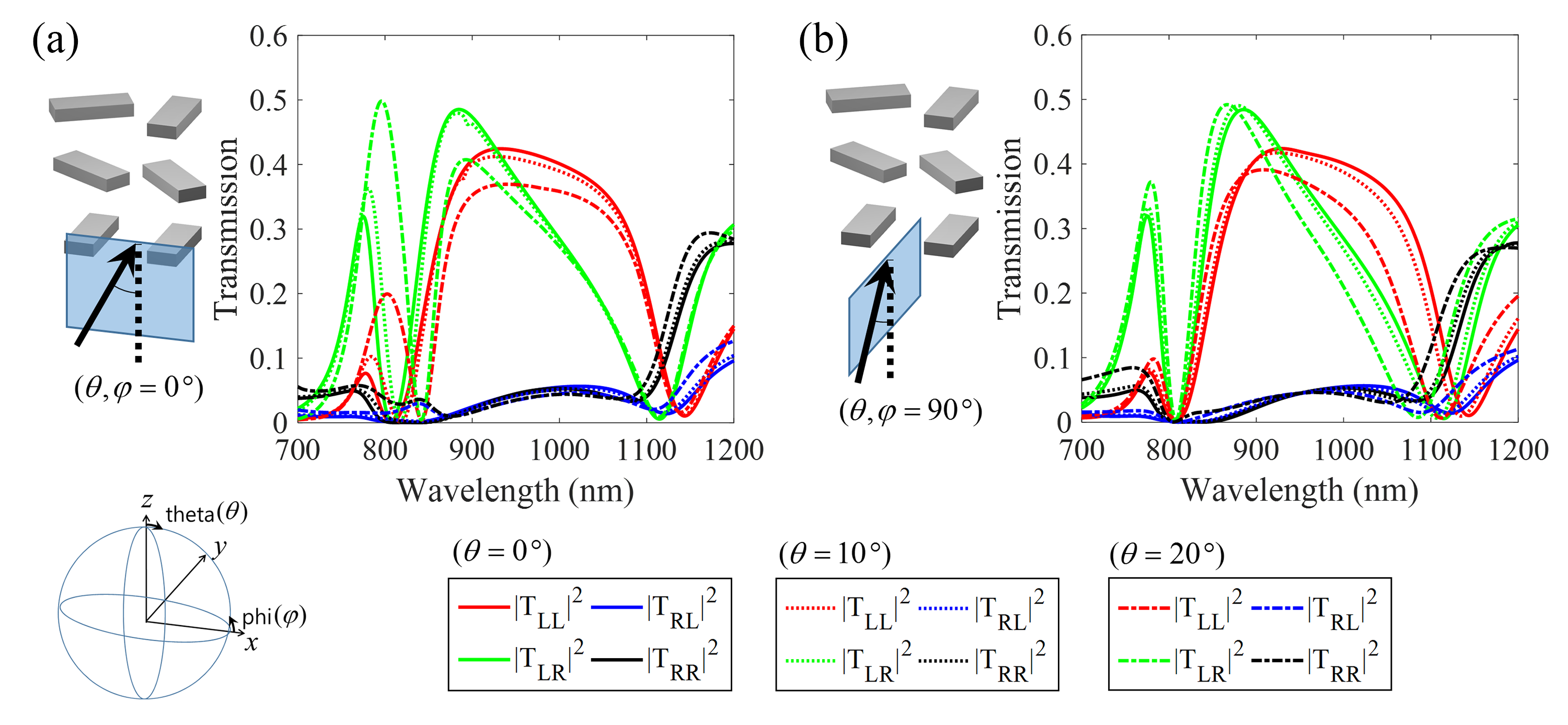


**Figure S4.** Transmission spectra of the designed metasurface for the different incident angles, *θ*, with (a) *φ* = 0 ° and (b) *φ* = 90 °. The spherical axis and legends below are the same for all spcetra.

References

[1] Grady, N. K. *et al.* Terahertz metamaterials for linear polarization conversion and anomalous refraction. *Science* **340**, 1304–1307 (2013).

[2] Liu, J. *et al.* High-efﬁciency mutual dual-band asymmetric transmission of circularly polarized waves with few-layer anisotropic metasurfaces. *Adv. Opt. Mater.* **4**, 2028–2034 (2016).
